# Lysosomal vulnerability as a therapeutic target in thyroid cancer using fucoidan nanoparticles

**DOI:** 10.1038/s41598-026-52121-6

**Published:** 2026-05-12

**Authors:** Marilena Celano, Agnese Gagliardi, Raffaella Gallo, Elena Giuliano, Diego Russo, Donato Cosco, Giuseppe Fiume

**Affiliations:** 1 Department of Health Sciences, University of Catanzaro “Magna Graecia”, Campus Universitario “S. Venuta”, Catanzaro, 88100 Italy; 2 Department of Experimental and Clinical Medicine, University of Catanzaro “Magna Graecia”, Campus Universitario “S. Venuta”, Catanzaro, 88100 Italy

**Keywords:** Colloids, Fucoidan, Human thyroid carcinoma, Lysosomes, Cathepsin D, Biochemistry, Biotechnology, Cancer, Drug discovery

## Abstract

Thyroid cancer represents the ninth most common malignancy worldwide, and a subset of cases exhibits aggressive behaviour with limited therapeutic options. Fucoidan, a sulphated polysaccharide, has demonstrated anticancer activity with an undefined mechanism. Here, we investigated the biological effects of fucoidan (FU) and fucoidan nanoaggregates (NFU) in both thyroid cancer (SW1736) and non-tumorigenic thyroid cells (Nthy-ori 3.1). NFU showed a mean diameter of ~ 187 nm, a low polydispersity (~ 0.2) and a negative Zeta potential. NFU significantly reduced SW1736 cell viability, reaching ~ 60% inhibition at 0.01 mg/mL and ~ 80% at 0.1 mg/mL after 72 h, while sparing non-tumorigenic cells. Mechanistically, NFU induced marked lysosomal enlargement selectively in tumor cells and localized predominantly within LAMP-1–positive compartments. Consistently, tumor cells showed constitutive Cathepsin D maturation and enhanced sensitivity to NFU-induced lysosomal perturbation. Collectively, these findings identify lysosomes as a primary intracellular target of fucoidan-based nanosystems and reveal a tumor-selective vulnerability to NFU-mediated lysosomal stress. This study provides the first quantitative evidence of lysosomal targeting by fucoidan nanoparticles in thyroid cancer cells and supports NFU as a promising lysosome-directed nanotherapeutic strategy for aggressive thyroid malignancies.

**Supplementary Information:**

The online version contains supplementary material available at 10.1038/s41598-026-52121-6.

## Introduction

Thyroid cancer represents the most common endocrine malignancy, with a rising incidence globally over recent decades. While differentiated thyroid carcinomas generally have favorable prognoses, undifferentiated subtypes such as anaplastic thyroid carcinoma (ATC) remain highly aggressive and resistant to conventional therapies based on surgery and radioiodine, underscoring the need for novel targeted treatment strategies^1^. The genetic and epigenetic characterization of thyroid tumors exerted an important role on the identification of novel molecular targets and currently, several combination of checkpoint inhibitors with other treatment approaches are currently being investigated. An innovative approach as the adoptive immunotherapy as the Anti-CD19 chimeric antigen receptor T cell (CAR-T) and has recently even been tested against preclinical models of thyroid cancer^2^. Unfortunately at present, none of the available treatments are satisfactorily effective for ATC. Clinical trial results indicate that the current targeted compounds are able to prolong progression-free survival rather than offer a decisive cure, because both the presence of intolerable adverse events and the occurrence of secondary resistance make this approach^3–5^. Therefore, the search for alternative therapies has increasingly focused on naturally derived bioactive compounds with selective anticancer properties and favorable safety profiles. In recent years, the exploration of bioactive molecules derived from natural sources as macroalgae rich in polysaccharides (e.g., fucoidans and alginates), polyphenols (e.g., phlorotannins), and terpenoids, has gained particular interest^6^. Fucoidan (FU) is a sulfated polysaccharide mainly derived from brown algae such as *Fucus vesiculosus*, *Undaria pinnatifida*, and *Laminaria japonica*, and has attracted growing interest due to its broad biological activities, including anticoagulant, antioxidant, immunomodulatory, and particularly antitumor effects^6–10^.

Extensive in vitro and in vivo studies have demonstrated that FU inhibits tumor cell proliferation and promotes apoptosis in multiple cancer types, including leukemia, breast, liver, and colon cancers^11–16^. The mechanisms underlying these effects are multifaceted, involving modulation of cell cycle progression, activation of caspase-dependent apoptotic pathways, and inhibition of angiogenesis and metastasis^17–19^. FU also interferes with tumor cell migration and adhesion by downregulating matrix metalloproteinases (MMPs) and vascular endothelial growth factor (VEGF), both key factors in metastatic progression^20–22^. Moreover, it also modulates several key oncogenic signaling pathways frequently altered in cancer, such as PI3K/Akt, MAPK, and mTOR, thus impacting cell survival, proliferation, and resistance to treatment^21^. Importantly, several studies have reported that FU not only inhibits tumor progression but also enhances the efficacy of conventional anticancer therapies^17^. In addition to its direct antitumor effects, FU improves the therapeutic response to standard treatments by altering the tumor microenvironment, increasing cancer cell susceptibility to chemotherapeutics, and reducing systemic toxicity^17,23,24^. One of the main challenges limiting the clinical translation of FU is its poor bioavailability, due to its high molecular weight, hydrophilicity, and limited cellular uptake^25^. To address these limitations, nanotechnology-based formulations such as FU-loaded nanoparticles have been developed. with the aim of modulating the bioavailability of the polysaccharide and enabling the targeted delivery to tumor tissues via the enhanced permeability and retention (EPR) effect^26,27^. Moreover, fucoidan nanoparticles showed synergistic effects when combined with standard chemotherapeutic agents, reducing the required drug dose and minimizing systemic toxicity^28^. In addition to its antiproliferative effects, emerging evidence suggests that FU may modulate intracellular organelles activities, including those of the lysosomal compartment^29,30^. Lysosomes play a central role in cellular homeostasis, acting as key regulators of autophagy, degradation of macromolecules, and metabolic signaling. Cancer cells frequently exhibit alterations in lysosomal function, including increased biogenesis, enzyme activity, and size, which may render them particularly susceptible to agents that further impair lysosomal homeostasis^31–35^. In cancer cells, lysosomes are not only more numerous but also functionally reprogrammed to support enhanced metabolic demands, promote invasion, and modulate cell death pathways^36–38^. This reprogramming includes alterations in lysosomal positioning, enzyme activity, pH regulation, and membrane stability, often contributing to cancer cell survival under stress conditions such as hypoxia, nutrient deprivation, or therapy-induced damage. Moreover, lysosomal stress has been implicated in the regulation of the mechanistic target of rapamycin complex 1 (mTORC1) pathway, which integrates nutrient sensing with cell growth and survival signals. Disruption of lysosomal function can inhibit mTORC1 signaling, thereby suppressing anabolic processes and sensitizing cancer cells to metabolic stress. In parallel, lysosomal dysfunction may impair autophagic flux, a process upon which many cancer cells rely for survival in hostile microenvironments^36–38^. Given the heightened vulnerability of cancer cells to lysosomal stress, therapeutic strategies that target lysosomal function are gaining attention as promising anticancer approaches^39^. In this context, we hypothesized that the selective cytotoxicity of FU and NFU toward thyroid cancer cells may be mechanistically linked to their impact on the lysosomal compartment. In this study, we investigated the antiproliferative effects of FU and NFU in both non tumorigenic (Nthy-ori 3 − 1) and anaplastic thyroid carcinoma (SW1736) cell lines. We observed a preferential cytotoxicity of both compounds in tumor cells, with stronger efficacy exerted by NFU at lower concentrations. Mechanistically, we show that these effects are associated with pronounced alterations in lysosomal morphology, suggesting that lysosomal dysfunction may underlie the enhanced vulnerability of thyroid cancer cells to these treatments. Our findings highlight a novel lysosome-targeted mechanism of action for fucoidan-based therapies and support the potential of NFU as a conceivable innovative drug delivery system to be employed for the treatment of aggressive thyroid malignancies.

## Results

### Physico-chemical characterization and morphological analysis of fucoidan nanoparticles

**Fig. 1:**
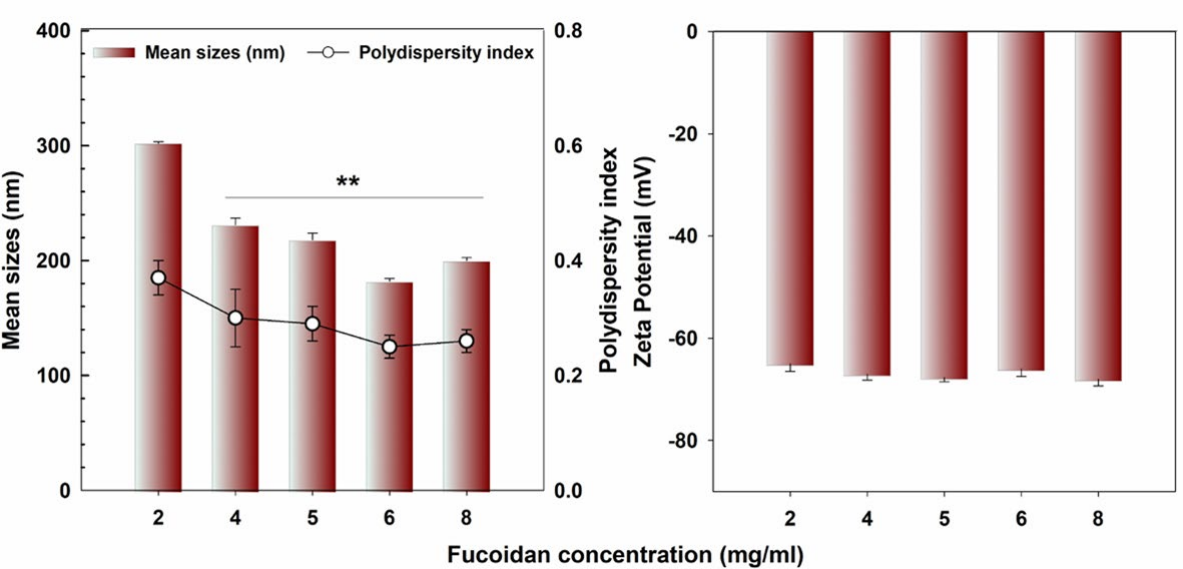
Physico-chemical characterization. Mean sizes, polydispersity index and Zeta potential of fucoidan nanoparticles as a function of the amount of the polysaccharide employed during the sample preparation. **p < 0.001 with respect to the mean sizes of the formulation prepared with 2 mg/ml of fucoidan.

Comprehensive physicochemical characterization, including particle mean diameter, size distribution, and Zeta potential, is pivotal for understanding and predicting the biological fate and in vivo performance of nanoparticles. These properties critically affect biodistribution, cell uptake, and interactions with biological membranes, and are particularly important for nanoparticles proposed for systemic administration and developed to be accumulated in specific body compartments. As shown in Fig. 1 an increase in fucoidan concentration from 2 to 6 mg/mL led to a progressive reduction in both the average particle size and the polydispersity index, indicating the formation of smaller nanosystems characterized by a narrow size distribution (Figs.S1-S5). A slight increase in both parameters was observed when a concentration of 8 mg/mL of FU was employed, suggesting a plausible onset of polymer saturation during the rearrangement of the polysaccharide (Fig. S5). The Zeta potential remained consistent across all concentrations, suggesting that the surface charge and thus colloidal stability was not notably influenced by changes in fucoidan content. Based on this analysis, the formulation prepared using 6 mg/mL of FU was selected for additional studies as a consequence of its favorable physico-chemical characteristics. The morphology of these nanosystems was investigated by TEM and it was shown a well-defined spherical structure characterized by a mean diameter lower than 200 nm, confirming the results obtained by DLS (Fig. S6).

**Fig. 2:**
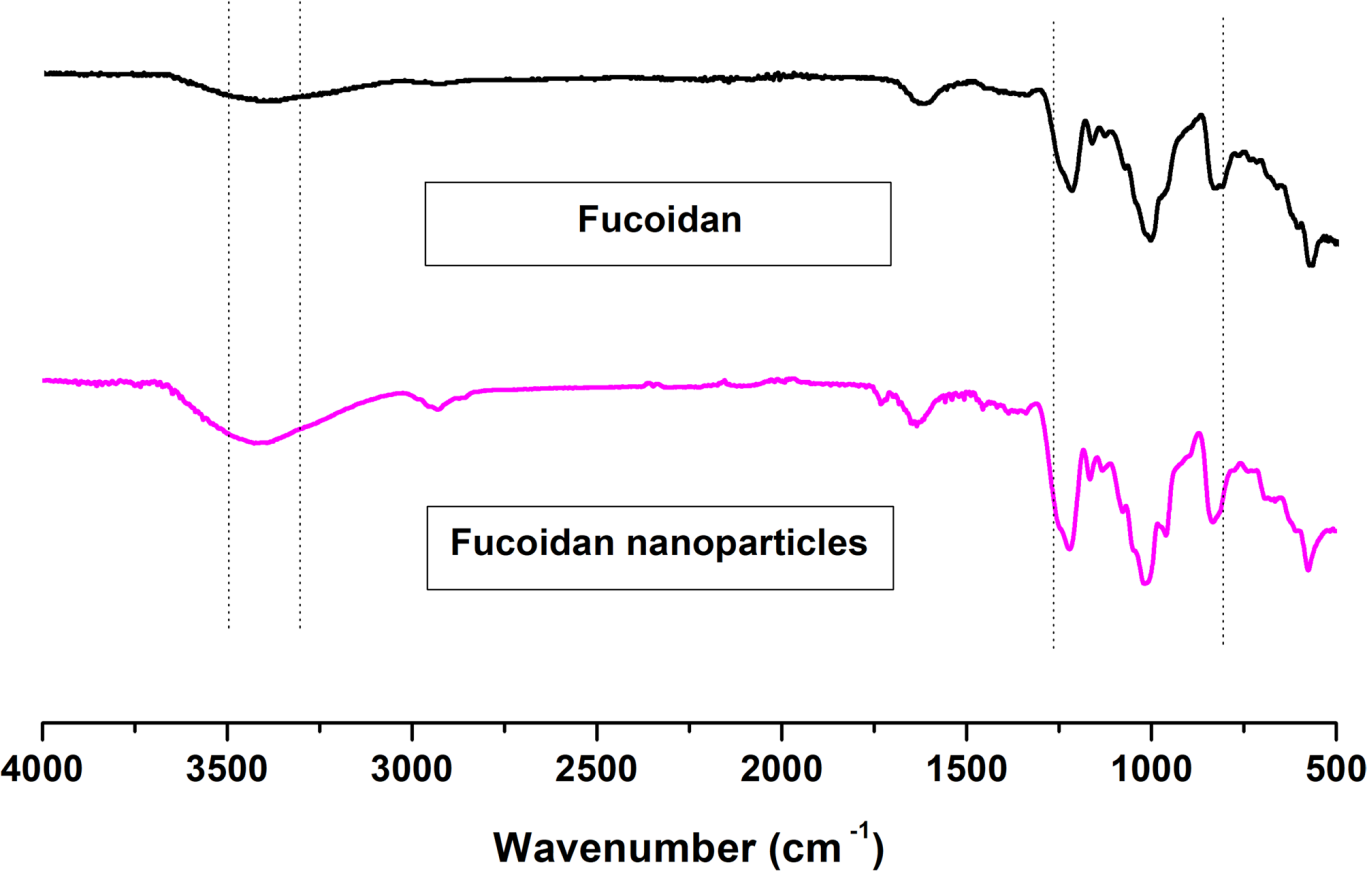
FT-IR spectra of fucoidan and fucoidan nanoparticles.

FT-IR spectroscopy was employed to compare fucoidan powder and freeze-dried fucoidan nanoparticles and to evaluate whether the formation of the colloidal formulation affected the chemical structure of the polysaccharide (Fig. 2). In the FT-IR spectrum of fucoidan powder, a broad absorption band in the 3200–3400 cm⁻¹ region is observed and attributed to O–H stretching vibrations of the polysaccharide backbone. In the fingerprint region, absorption contributions around 1250–1220 cm⁻¹ are associated with S = O stretching vibrations of sulfate ester groups, while bands observed at approximately 840–820 cm⁻¹ correspond to C–O–S vibrations characteristic of sulfated polysaccharides.

The FT-IR spectrum of the lyophilized fucoidan nanoparticles closely resembles that of fucoidan powder with preservation of the O–H stretching band and the sulfate-associated regions. Little variations in band intensity and band broadening are observed, which may be related to changes in intermolecular interactions and molecular organization upon the formation of the colloidal structure and freeze-drying process. Importantly, no loss of sulfate-related bands or appearance of new absorption peaks is observed, indicating that the obtained formulation does not compromise the chemical structure of fucoidan.

### Selective antiproliferative effects of fucoidan formulations and associated lysosomal alterations in thyroid cancer cells

**Fig. 3:**
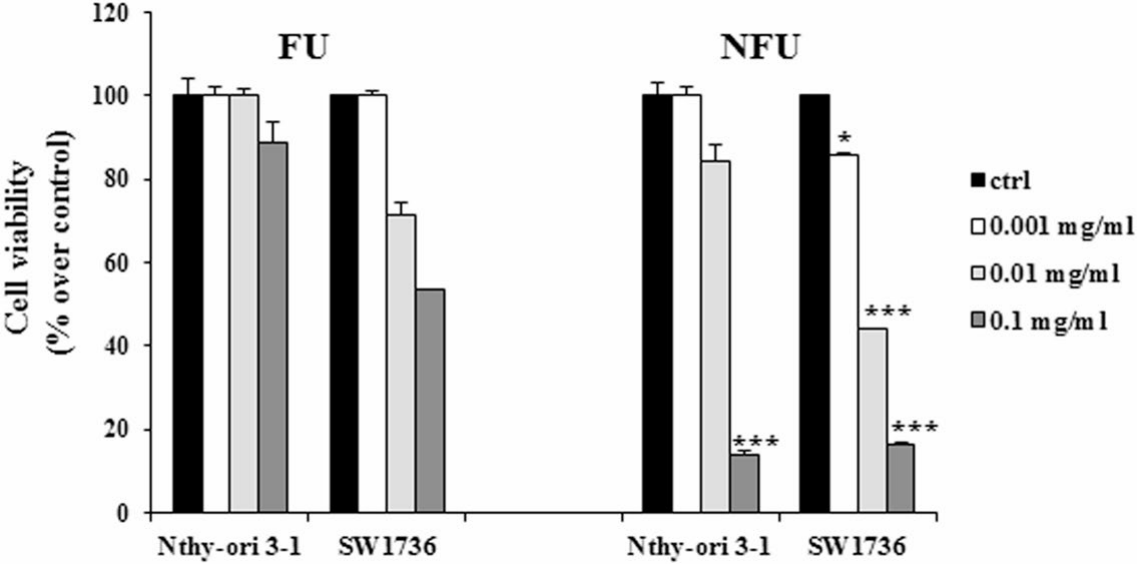
Antiproliferative effects of free Fucoidan and its nanoparticle-based formulation on Nthy-ori 3.1 and SW1736 cells. Cell viability was assessed using the MTT assay after 72 h of treatment. Results are expressed as a percentage relative to untreated control cells (ctrl) and are presented as mean ± standard error. *p < 0.05; ***p < 0.001 vs. ctrl. Fucoidan and fucoidan nanoparticles are indicated as FU and NFU, respectively.

Next, we tested the effects of FU and NFU on the viability of anaplastic thyroid cancer cells and non-tumorigenic cells. After 72 h of treatment, a reduction of cell growth was evident only in SW1736 cells with FU 0.01 and 0.1 mg/ml (~ 30% and 50% vs. untreated cells, respectively) (Fig. 3). A stronger significant reduction of viability was observed in SW1736 treated with NFU at all doses (0.001 mg/ml ~ 20%, *p* < 0.05; 0.01 ~ 60%, *p* < 0.001; ~80%, *p* < 0.001 vs. untreated cells) (Fig. 2). On the other hand, did not cause a significant change in cell viability with a non tumorigenic thyroid cells Nthy-ori 3 − 1.

**Fig. 4:**
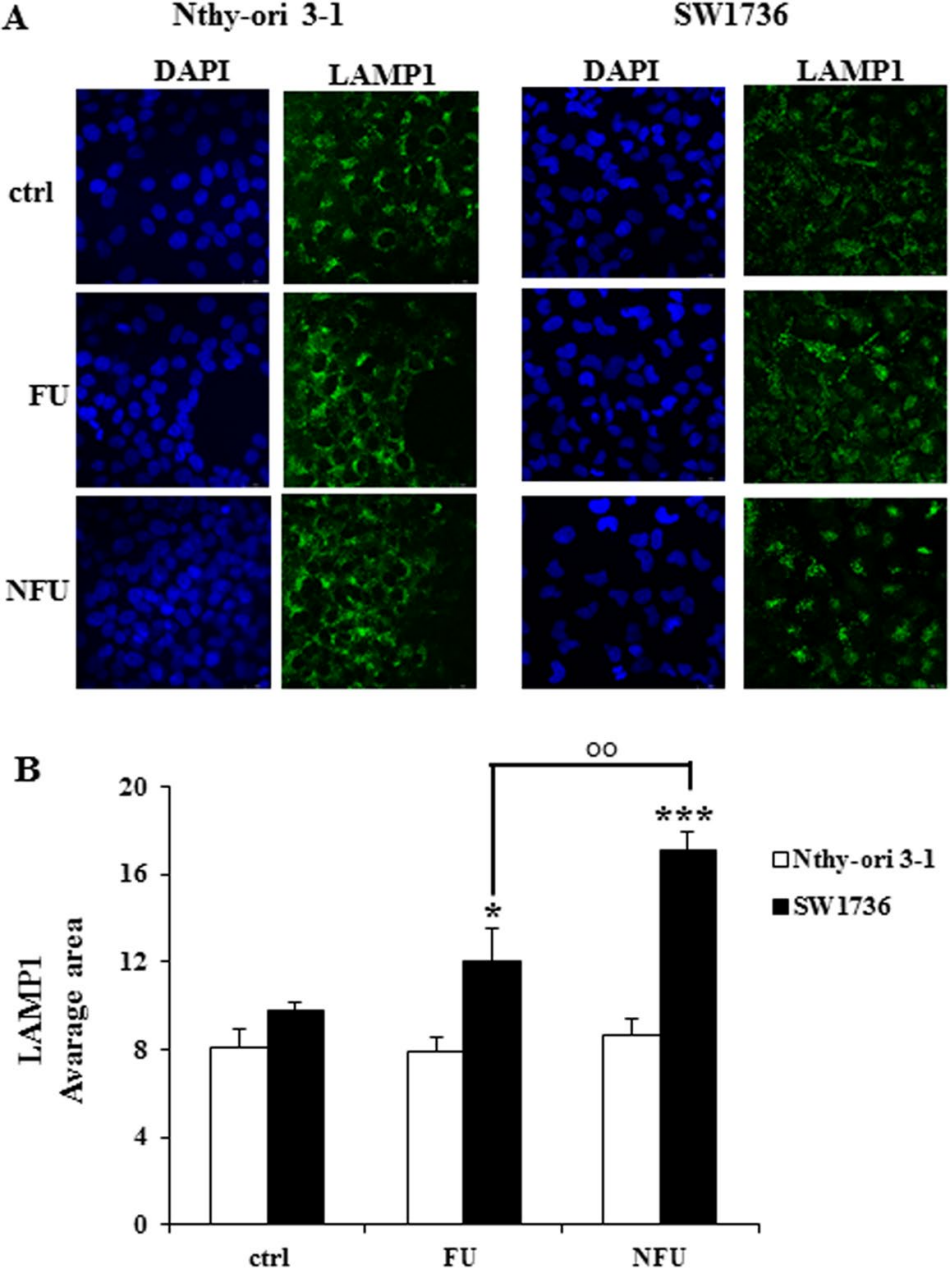
Effects of free Fucoidan and its nanoparticle-based formulation on lysosomes. (**A**) Representative immunofluorescence images of LAMP1 (green) showing lysosomal distribution; cell nuclei were stained with DAPI (blue). (**B**) Quantification of LAMP1-positive lysosomal area in SW1736 and Nthy-ori 3 − 1 cells. Results are expressed as the average of LAMP1-positive lysosomal area and presented as mean ± standard error. *p < 0.05; ***p < 0.001 vs. ctrl; °°*p* < 0.01 vs. FU. Fucoidan and fucoidan nanoparticles are indicated as FU and NFU, respectively.

Given our initial observations showing that both fucoidan and NFU reduced cell viability more effectively in anaplastic thyroid carcinoma cells than in non tumorigenic thyroid cells (Fig. 3), we sought to investigate the potential intracellular pathways affected by these treatments. In particular, we aimed to explore whether the lysosomal compartment, a key regulator of cellular homeostasis and autophagy, could represent a differential target of fucoidan and nanofucoidan in normal versus cancerous thyroid cells. To this end, we analyzed lysosomal morphology and autophagic markers through immunofluorescence staining of LAMP1 under basal conditions and following treatment. Under basal conditions, SW1736 tumor cells displayed a markedly increased lysosomal size compared to normal Nthy-ori 3-1cells, as indicated by a significant enlargement in LAMP1-positive area (Fig. 4). This observation suggests an accumulation of enlarged and potentially dysfunctional lysosomes, possibly due to impaired lysosomal clearance or excessive autophagic flux burden. The treatment of SW1736 cells with either fucoidan or nanofucoidan further exacerbated this lysosomal enlargement, in particular with NFU inducing a more pronounced effect than fucoidan alone (Fig. 4). These findings suggest that fucoidan-based treatments modulate lysosomal homeostasis in tumor cells, potentially enhancing lysosomal stress or interfering with lysosomal turnover mechanisms.

### Lysosomal targeting and intracellular accumulation of NFU in thyroid cancer cells

**Fig. 5:**
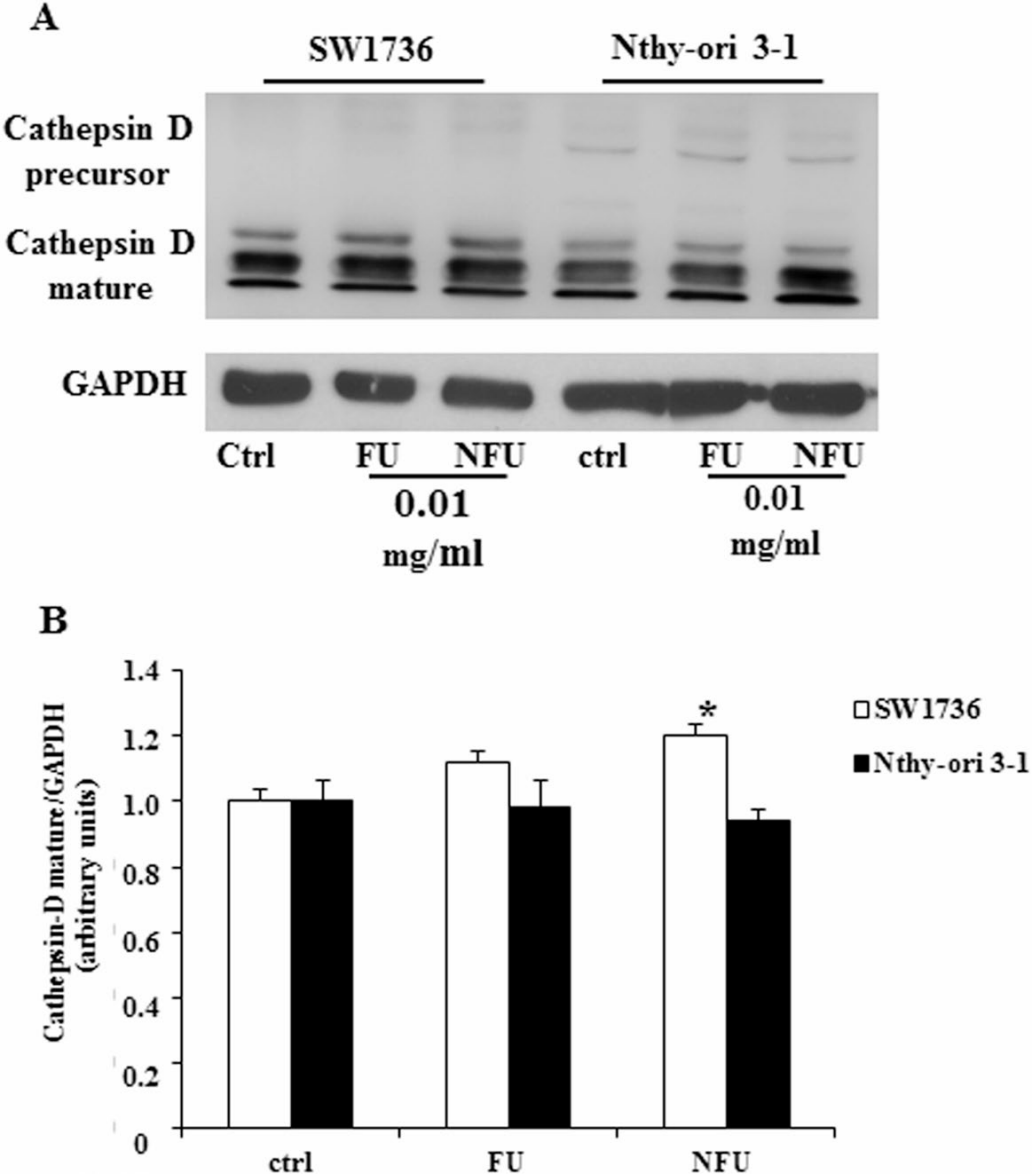
Effects on Cathepsin D. **A** Immunoblot analysis of Cathepsin D in SW1736 and Nthy-ori 3 − 1 cells after fucoidan (FU) and fucoidan nanoparticles (NFU) treatment. GAPDH was used as loading control. **B** Densitometric analysis from a representative immunoblot of Cathepsin D. Values are expressed as a ratio over the control (arbitrarily assigned as 1).

**Fig. 6:**
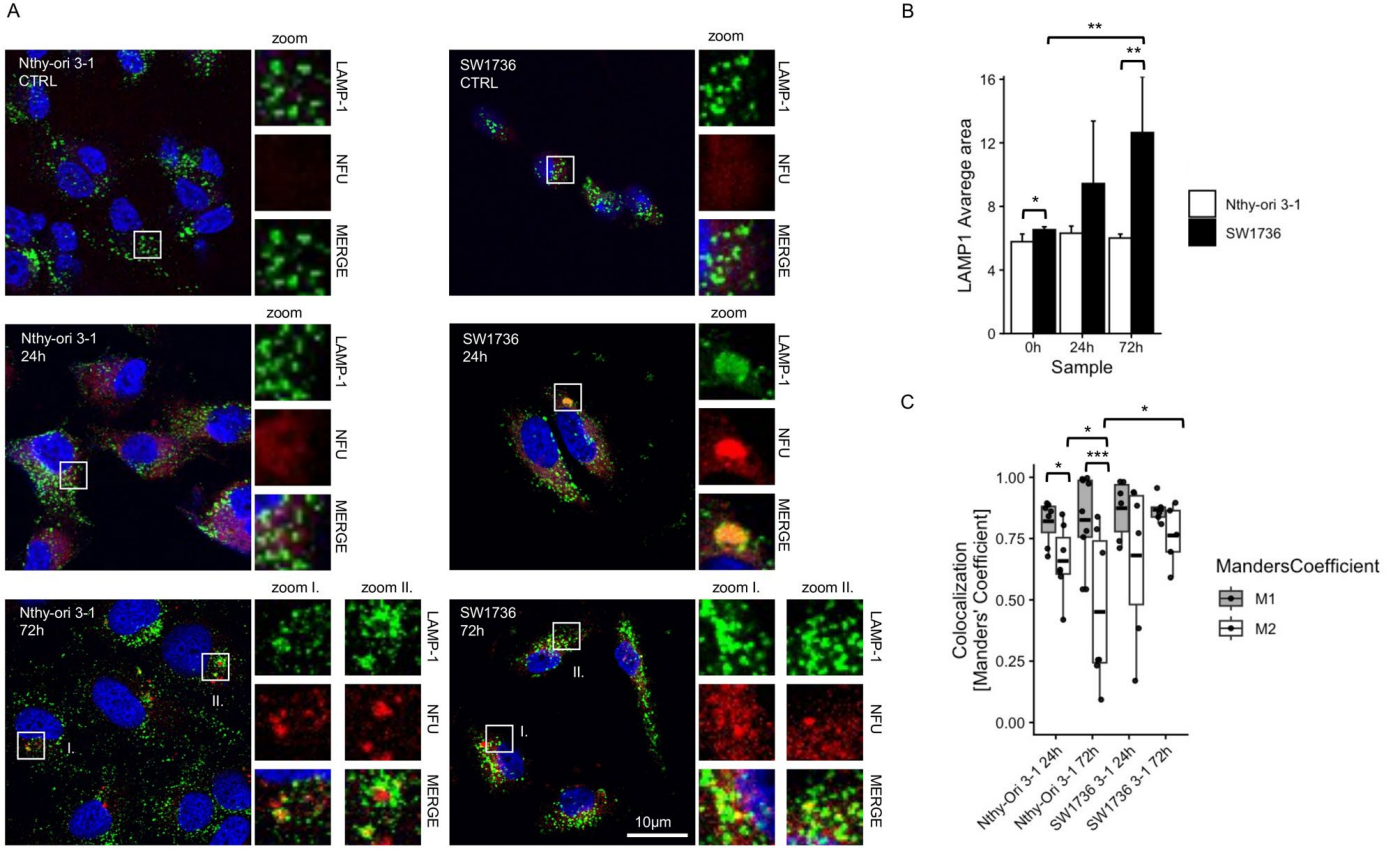
NFU uptake and lysosomal colocalization in thyroid cells. Intracellular trafficking and lysosomal localization of rhodamine-labeled NFU in anaplastic thyroid cancer (SW1736) and non-tumorigenic thyroid (Nthy-ori 3 − 1) cells. (**A**) Representative confocal microscopy images of SW1736 and Nthy-ori 3 − 1 cells treated with rhodamine-labeled NFU for 24 h and 72 h and immunostained for LAMP-1. NFU (red), LAMP-1 (green), and nuclei (blue). Merged images show extensive colocalization of NFU with LAMP-1–positive compartments (yellow). Scale bar: 10 μm. (**B**) Quantification of LAMP-1–positive area showing progressive lysosomal enlargement, significantly more pronounced in SW1736 cells compared to Nthy-ori 3 − 1 cells following NFU treatment. (**C**) Quantitative colocalization analysis between NFU and LAMP-1 using Manders’ coefficients. M1 represents the fraction of NFU signal overlapping LAMP-1, while M2 represents the fraction of LAMP-1 signal overlapping NFU. Data show efficient lysosomal targeting of NFU in both cell lines, with SW1736 cells displaying increased persistence of NFU within lysosomes at 72 h. Data are presented as mean ± SD. Statistical significance was assessed using Student’s *t* test; *p* < 0.05, *p* < 0.01, *p* < 0.001.

To gain further mechanistic insights into lysosomal function and its potential contribution to the observed cytotoxicity, we analyzed the expression of Cathepsin D, a lysosomal aspartic protease whose precursor (52 kDa) is proteolytically processed in the acidic environment of lysosomes to produce an active mature form (~ 34 kDa) (Fig. 5). After treatment with FU and NFU, interestingly, we found that the precursor form of Cathepsin D was detectable only in normal Nthy-ori 3 − 1 cells, whereas it was absent in untreated or treated SW1736 cells (tumoral thyroid cell line), where only the mature form was observed (Fig. 5A). This suggests that Cathepsin D undergoes more efficient or accelerated maturation in SW1736 cells, possibly reflecting a constitutive activation or stress-related remodeling of the lysosomal system. The absence of the precursor form in tumor cells may also indicate a shift toward increased lysosomal proteolytic processing, which is often observed in cancer cells undergoing metabolic or autophagic reprogramming. Moreover, treatment with FU or NFU led to a slight increase in the mature form of Cathepsin D specifically in SW1736 cells, with NFU inducing a more pronounced effect than fucoidan alone, but not in Nthy-ori 3-1cells, (Fig. 5). This observation reinforces the hypothesis that the lysosomal system in tumor cells is both altered at baseline and highly sensitive to additional perturbations, such as those induced by Fucoidan-based treatments. The combination of constitutive lysosomal activation and drug-induced lysosomal stress may overwhelm the system, leading to functional collapse and cell death, thus explaining the selective antiproliferative effects observed in tumor cells. SW1736 and Nthy-ori 3 − 1 cells were treated with rhodamine-labeled NFU for 24, 72 h or left untreated, and subsequently stained for the lysosomal marker LAMP-1 to investigate the intracellular fate of NFU and their relationship with lysosomes. Confocal microscopy revealed progressive intracellular accumulation of NFU in punctate structures and a concomitant enlargement of LAMP-1–positive compartments, which was markedly more pronounced in SW1736 cells compared to Nthy-ori 3 − 1 cells (Fig. 6A, B). NFU particles were predominantly detected in LAMP-1-positive structures after 72 h incubation (Fig. 6A). In SW1736 cells, lysosomal enlargement over time was accompanied by increased accumulation of NFU within LAMP-1–positive compartments (Fig. 6A, C), suggesting sustained lysosomal trafficking and retention. To quantitatively assess NFU–lysosome colocalization, Manders’ coefficients were evaluated. Given the punctate distribution of NFU and the heterogeneous size and intensity of lysosomes, Manders’ coefficients were considered the most appropriate metric to evaluate biologically relevant overlap. Manders’ coefficient M1, representing the fraction of NFU signal overlapping LAMP-1–positive compartments, was consistently high in both cell lines at all time points analyzed. In SW1736 cells, M1 values were 0.87 ± 0.12 at 24 h and 0.87 ± 0.06 at 72 h. Similarly, in Nthy-ori 3 − 1 cells, M1 values were 0.82 ± 0.18 at 24 h and remained elevated at 72 h (0.82 ± 0.09) (Fig. 6C). These results indicate that the majority of intracellular NFU localizes to LAMP-1–positive compartments, identifying lysosomes as the primary intracellular destination of NFU in both cancerous and non-cancerous thyroid cells. Manders’ coefficient M2, representing the fraction of LAMP-1 signal overlapping NFU, showed greater variability between cell lines and time points. In Nthy-ori 3 − 1 cells, M2 values were significantly lower than M1 at both 24 h (0.68 ± 0.15) and 72 h (0.45 ± 0.31), suggesting that while most NFU localizes to lysosomes, a substantial fraction of lysosomes does not retain NFU over time, possibly reflecting clearance or redistribution mechanisms. In contrast, SW1736 cells exhibited M2 values of 0.68 ± 0.33 at 24 h and 0.76 ± 0.12 at 72 h, with no significant difference between M1 and M2. Notably, M2 values at 72 h were significantly higher in SW1736 compared to Nthy-ori 3 − 1 cells, indicating that a larger proportion of lysosomes remains associated with NFU at later time points in tumor cells. Collectively, these data demonstrate efficient uptake and lysosomal trafficking of NFU in both cell lines. Lysosomal targeting occurs early after internalization and is maintained over time. However, the extent and persistence of lysosomal association, as well as the degree of lysosomal enlargement, differ markedly between normal and cancer cells, with SW1736 cells displaying enhanced lysosomal accumulation of NFU and more pronounced lysosomal remodeling.

## Discussion

To date, many clinical trials on ATC, one of the rarest and most aggressive thyroid malignancies and characterized by prognosis dismal, are ongoing^1^. ATC account for only 1–2% of all thyroid cancer cases, but the disease-specific mortality rate is close to 100%. For this reason, although the results of treatments improving year by year^2^, the mortality rate limited the availability of prospective trial results, therefore no standard chemotherapeutic option for unresectable or metastatic anaplastic thyroid cancer has yet been established. The anticancer treatment options available are: cytotoxic agents, molecular target agents, immune checkpoint inhibitors and their combinations. In the last years, moreover, several kinase inhibitor combinations have been approvals for the treatment but a possible mechanism of acquired resistance and the onset side effects limits its use, therefore is necessary to identify new targets and drugs^1,32^.

In the last years, seaweeds demonstrated to be a valuable natural source of polysaccharides characterized by several biological activities^40^. Among them, fucoidan was investigated as a consequence of its anti-inflammatory and antitumor properties and it is usually uptaken into the cells through endocytosis and macropinocytosis and it is localized within lysosomes via tubulin pathway, promoting autophagy and reducing lipid accumulation in foam cells^29,30^.

Lysosomes are involved in cellular metabolism, cell proliferation and differentiation, immunity, and cell death, and in recent years several studies have demonstrated the correlation between lysosomal change and disfunction with development disease such as cancer^32^.

In this study, we demonstrate that FU and its colloidal form NFU exert selective antiproliferative effects against anaplastic thyroid carcinoma (SW1736) cells while sparing non-tumoral thyroid cells (Nthy-ori 3 − 1) (Fig. 3). Notably, NFU was more potent than the free form of the polysaccharide^41^, reducing cell viability at lower concentrations. This differential sensitivity led us to investigate whether intracellular organelle homeostasis, specifically that of lysosomes, might contribute to the observed phenomena. Once considered static recycling centers for cellular waste, lysosomes are now recognized as highly dynamic organelles that integrate metabolic signals and regulate the balance between anabolic and catabolic processes^42^. Recent discoveries have revealed that lysosomes serve as key hubs for sensing environmental and nutrient cues, orchestrating cellular responses through coordinated regulation of autophagy, lysosomal biogenesis, and metabolic adaptation^42–44^. Moreover, lysosomes engage in extensive crosstalk with other organelles, forming membrane contact sites and mediating vesicular trafficking and content exchange. Their spatial positioning within the cell, once thought incidental, is now known to critically influence their function. In light of this broader understanding, lysosomal dysfunction is emerging as a unifying feature not only of rare lysosomal storage diseases, but also of more prevalent conditions such as neurodegenerative, and neoplastic disorders. In cancer, in particular, dysregulated lysosomal activity supports enhanced autophagy, promotes survival under stress, and contributes to therapy resistance, making the lysosomal compartment a compelling and actionable therapeutic target. Our results reveal that SW1736 cells exhibit an increased basal lysosomal area, as assessed by LAMP1 immunofluorescence, in comparison to normal Nthy-ori 3 − 1 cells (Fig. 4). This morphological phenotype likely reflects a pre-existing lysosomal impairment or overload in tumor cells. Enlarged lysosomes are commonly observed in cancer cells^45^ and are often indicative of altered lysosomal turnover, excessive autophagic flux, or defective lysosomal clearance. Indeed, lysosomal enlargement is a well-established marker of cellular stress and has been linked to reduced degradative efficiency and accumulation of undegraded material, both of which compromise lysosomal functionality^46^. Importantly, the use of the fucoidan formulations further exacerbated lysosomal enlargement in SW1736 cells but not in normal cells, suggesting that these compounds selectively disrupt lysosomal homeostasis in cancer cells. The more pronounced effect observed with NFU supports the idea that the colloidal conformation of the polysaccharide may enhance the lysosomal accumulation of the biomolecule, thereby amplifying its biological effects. Such exacerbation of lysosomal stress may push an already compromised lysosomal system beyond a functional threshold, resulting in activation of cell death pathways. Lysosomes are not passive organelles but dynamic regulators of survival, metabolism, and death; thus, their destabilization can lead to lysosomal membrane permeabilization, release of cathepsins, and caspase activation, hallmarks of lysosomal-mediated cell death^47–49^. Further supporting the notion of lysosomal dysfunction, we observed distinct differences in the processing of Cathepsin D, a key lysosomal aspartic protease (Fig. 5). While both precursor and mature forms of Cathepsin D were present in normal Nthy-ori 3 − 1 cells, only the mature form was detectable in SW1736 cells, even under untreated conditions. This suggests a constitutively enhanced lysosomal proteolytic activity in tumor cells, potentially as a compensatory adaptation to increased metabolic and autophagic demands. The absence of the precursor form may also indicate dysregulation of protease trafficking or processing, as seen in other malignancies with altered lysosomal function^50,51^. Interestingly, both FU and NFU led to a further increase in the mature form of Cathepsin D in SW1736 cells but not in Nthy-ori 3 − 1 cells. This selective upregulation likely reflects an attempt by tumor cells to cope with exacerbated lysosomal stress by increasing proteolytic activity. However, this compensatory response may be insufficient or even detrimental, ultimately tipping the balance toward cell death. The fact that NFU induced a more pronounced effect is consistent with its greater impact on lysosomal morphology and antiproliferative activity. Taken together, our findings suggest that the cytotoxic effects of fucoidan-based formulations in thyroid cancer cells are mediated, at least in part, by disruption of lysosomal homeostasis. Tumor cells, which already exhibit basal lysosomal alterations, appear to be more vulnerable to further lysosomal stress induced by these compounds. This vulnerability may be therapeutically exploitable, as selective lysosomal destabilization in cancer cells represents an emerging anticancer strategy. Agents that interfere with lysosomal function have shown promise in preclinical models, particularly in tumors and neurodegenerative disorders with heightened autophagic or lysosomal activity^52–54^. To further clarify the intracellular fate of NFU and strengthen the evidence for lysosomal targeting, we performed confocal microscopy using rhodamine-labeled NFU combined with LAMP-1 immunostaining in both anaplastic thyroid cancer and non-tumorigenic thyroid cells (Fig. 6). These experiments revealed efficient internalization of NFU in both cell lines, followed by predominant trafficking to LAMP-1–positive compartments, identifying lysosomes as the primary intracellular destination of the nanoparticles. Quantitative colocalization analysis using Manders’ coefficients demonstrated consistently high overlap between NFU and lysosomes (M1 > 0.8), indicating that the majority of internalized NFU resides within lysosomal structures. Importantly, while lysosomal targeting occurred in both cell types, SW1736 cells displayed significantly greater lysosomal enlargement and increased persistence of NFU within LAMP-1–positive compartments at later time points compared to Nthy-ori 3 − 1 cells. This differential retention suggests impaired lysosomal clearance or altered vesicular trafficking in tumor cells, consistent with the presence of enlarged lysosomes observed under basal conditions. Such intrinsic lysosomal remodeling is a recognized feature of aggressive cancer phenotypes and may render tumor cells particularly vulnerable to additional lysosomal stress^36,43,44^. These findings provide direct visual and quantitative evidence that NFU accumulate within lysosomes and exacerbate pre-existing lysosomal alterations in anaplastic thyroid cancer cells. Together with the observed constitutive maturation of Cathepsin D and the selective antiproliferative effects of NFU, our data support a model in which nano-formulated fucoidan acts as a lysosome-targeting agent that amplifies lysosomal dysfunction in tumor cells, ultimately exceeding their adaptive capacity and contributing to cell death. On the contrary, non-tumorigenic thyroid cells show reduced lysosomal retention of NFU and limited morphological remodeling, consistent with their resistance to NFU-induced cytotoxicity.

In conclusion, this study demonstrates for the first time to the best of our knowledge that fucoidan, particularly when formulated as nanoparticles, exerts selective antiproliferative effects against anaplastic thyroid cancer cells while sparing non-tumorigenic thyroid cells. We identify the lysosomal compartment as a primary intracellular target of nano-formulated fucoidan, showing that NFU are efficiently internalized and preferentially retained within LAMP-1–positive compartments. This lysosomal accumulation is associated with pronounced lysosomal enlargement and altered Cathepsin D processing in tumor cells, indicating exacerbation of pre-existing lysosomal dysfunction. Our findings support the concept that lysosomal vulnerability represents a therapeutically exploitable feature of aggressive thyroid cancers and that nanoparticle-based formulations can enhance organelle-specific targeting and biological efficacy. However, this study is limited to in vitro models, and the precise molecular events linking lysosomal perturbation to cell death were not directly addressed. In addition, functional assays of lysosomal integrity and autophagic flux were not performed and will be required to fully elucidate the downstream mechanisms involved. Despite these limitations, the present work provides direct quantitative evidence of lysosomal targeting by fucoidan nanoparticles and highlights their potential as a platform for the development of lysosome-directed nanomedicines. Future studies will be necessary to validate these findings in in vivo models and to assess the translational relevance of NFU-based strategies for the treatment of aggressive and therapy-resistant thyroid cancers.

## Methods

### Preparation of fucoidan nanoparticles

Fucoidan nanoparticles (NFU) were prepared using the antisolvent method, in which an organic solvent is added to an aqueous solution of the polymer to induce nanoprecipitation. Specifically, FU derived from *Fucus Vescicolosus* (purity ≥ 95%, ∼70 kDa) (Merck Life Science S.r.l., Milan, Italy) was dissolved in distilled water at concentrations ranging from 2 to 8 mg/mL. To each 5 mL aliquot of the aqueous fucoidan solution, 4 mL of acetone were added dropwise at a rate of 1.0 mL/min under constant magnetic stirring (600 rpm) at room temperature. The resulting dispersions were kept under stirring for 12 h to allow complete evaporation of the organic solvent and stabilization of the nanoparticle suspension. The nanoparticles were then purified by ultracentrifugation at 90,000 × g for 60 min at 4 °C. d. Fluorescent NFU was obtaining solubilizing rhodamine-DHPE (0.01% w/v) in the organic phase during the preparation procedure.

### Physico-chemical characterization of fucoidan nanoparticles

Dynamic light scattering (DLS) analysis was employed to evaluate the average hydrodynamic diameter, polydispersity index (PDI), and Zeta potential of NFU. Measurements were carried out using a Zetasizer Advance Pro (Malvern Panalytical Ltd., UK), based on the third-order cumulant fitting of the autocorrelation function. For each formulation, three independent batches were analyzed in triplicate, and the results were expressed as mean intensity (%) ± standard deviation.

The morphology of the nanosystems was investigated by transmission electron microscopy (CM12 TEM, PHILIPS, The Netherlands) equipped with an OLYMPUS Megaview G2 camera. Fourier-transform infrared (FT-IR) spectroscopy analysis was carried out using a NicoletTM iS5 spectrometer coupled with an iD7 Attenuated Total Reflectance accessory (Thermo Fisher Scientific Inc., Waltham, MA, USA). Spectral acquisition and processing were performed using OMNIC software (version 9.12.1019). All analyses were carried out in triplicate, and the reported spectra are representative of three independent experiments^55^.

### Cell culture

In this study, we have used an immortalized human thyroid cancer cell lines SW1736 characterized by the presence of BRAFV600E mutation and, as a control, we have used the human non-tumorigenic thyroid cell line Nthy-ori 3 − 1, widely adopted as a model of normal human thyroid cells (American Type Culture Collection; LGC Standards s.r.l., Milan, Italy)^53^. Short Tandem Repeat analysis was performed to check the genomic stability of these cell lines.

Cells were cultured in RPMI or DMEM (Thermo Fisher Scientific Inc., Waltham, MA, USA) containing 10% fetal bovine serum (Thermo Fisher Scientific Inc.) and supplemented with penicillin (100UI/ml) and streptomycin (0.1 mg/ml) and amphotericin B (2.5 µg/ml) (Sigma Aldrich S.r.l., Milan, Italy). Cells were cultured at 37° C in a humidified 5% CO_2_ atmosphere.

### Cell viability

Cell proliferation was evaluated by MTT assay performed in 96-well plates containing SW1736 and Nthy-ori 3 − 1 cells seeded at a density of 4 × 10^3^ and 6 × 10^3^ respectively. Twenty-four hours later when the cells had reached 60–80% confluence, fresh normal medium was supplemented with FU (Sigma Aldrich S.r.l., Milan, Italy) and NFU at different doses, (0.001, 0.01, 0.1 mg/ml). After 72 h of treatment, the cells were incubated with methyl thiazolyl tetrazolium (MTT) for 3 hours and crystals of formazan were quantified with a microplate spectrophotometer (VARIOSKAN LUX, Thermo Fisher Scientific Inc.,Waltham, MA, USA) at a wavelength of 540 nm and a reference wavelength of 690 nm. Results are expressed as percentages over untreated cells indicated as ctrl.

### Immunofluorescence assay

SW1736 and NTHY-ORI 3.1 cells were grown on coverslips in 6-well plates. After 72 h of incubation with FU and NFU 0.01 mg/ml, were fixed with 4% paraformaldehyde, permeabilized with Triton X-100 and processed for immunofluorescence as previously described^54,56,57^. Briefly, cells were immunostained with anti-LAMP1 antibody (Thermo Fisher Scientific Inc) diluted 1:500, and anti-P62 antibody (Santa Cruz Biotechnologies) diluted 1:300, and then incubated with Alexa Fluor 555 anti-mouse antibody from Molecular Probes (Thermo Fisher Scientific Inc.) and with Alexa Fluor 488 anti-rabbit antibody at 1:300 dilution. Cells were visualized under confocal microscope Leica Stellaris 5.

### NFU uptake and lysosomal co-localization

For NFU uptake and lysosomal colocalization experiments, SW1736 and Nthy-ori 3 − 1 cells were incubated with NFU for 0, 24, or 72 h, followed by fixation and immunofluorescence staining as described above and previously described^56,57^. Confocal images were acquired using rhodamine-labeled NFU, while lysosomes were visualized by LAMP-1 immunostaining using an Alexa Fluor 488–conjugated secondary antibody. Lysosomal size was quantified using Fiji (ImageJ) by applying an intensity threshold to generate binary masks of LAMP-1–positive structures. These masks were analyzed using the Analyze Particles function with the following parameters: particle size (pixel²) = 1–100 and circularity = 0.01–1.00. Colocalization between NFU and lysosomes was quantified using Manders’ overlap coefficients (M1 and M2), calculated with the JACoP plugin in Fiji. M1 represents the fraction of the NFU signal overlapping with LAMP-1–positive compartments, whereas M2 represents the fraction of the LAMP-1 signal overlapping with NFU. Prior to colocalization analysis, confocal images were automatically thresholded using the Costes method to exclude background signal. At least three independent fields of view were analyzed for each condition to account for both intracellular and intercellular variability.

### Western blot analysis

Thyroid cells were treated with FU and NFU 0,01 mg/ml and after 72 h of incubation, proteins were extracted as previously described^5,52^. Twenty µg of proteins were run on 12% SDS-PAGE gel, transferred to PVDF membranes (VWR, Milan, Italy), blocked with phosphate buffered saline, 0.1% Triton and 5% non-fat dry milk (PBS-T/milk) and incubated overnight with the following antibodies: monolonal anti-cathepsin D antibody diluted 1:500, (Santa Cruz Biotechnology, DBA, Segrate, Milan, Italy), anti-GAPDH (Sigma Aldrich, Milan, Italy) diluted 1:10.000 that was used as internal control. Then, the membranes were washed in PBS-T and incubated with horseradish peroxidase-conjugated anti-rabbit or anti-mouse antibody (Transduction Laboratories, Lexington, KY, USA). Western blot detection system ECL Plus (Perkin Elmer, Monza, Italy) was used to visualize proteins. Protein bands were quantified by ImageJ software.

### Statistical analysis

All results were analyzed by one-way ANOVA followed by the Tukey-Kramer multiple comparisons test using GraphPad Prism version 5.0 statistical software (GraphPad Software Inc., San Diego, CA, USA). The results are expressed as means ± standard error or standard deviation and *p* values lower than 0.05 were considered statistically significant.

## Supplementary Information

Below is the link to the electronic supplementary material.

Supplementary Material 1

## Data Availability

All data generated during this study are included in this published article.
