# Supplementary material for: Lysosomal vulnerability as a therapeutic target in thyroid cancer using fucoidan nanoparticles
Source: Sci Rep. 2026 May 12;16:21600. doi: 10.1038/s41598-026-52121-6 (PMC13350920; doi:10.1038/s41598-026-52121-6)
Supplement: Supplementary file 1 — Supplementary Material 1 [file 41598_2026_52121_MOESM1_ESM.pdf]

SUPPLEMENTARY INFORMATION

Lysosomal Vulnerability as a Therapeutic Target in Thyroid Cancer Using Fucoidan Nanoparticles

Marilena Celano[1,+,*], Agnese Gagliardi[1,+], Raffaella Gallo[2], Elena Giuliano[1], Diego Russo[1], Donato Cosco[1,*] and Giuseppe Fiume[2,*]

[1]Department of Health Sciences and [2]Department of Experimental and Clinical Medicine University of Catanzaro “Magna Græcia”, Campus Universitario “S. Venuta”, I-88100 Catanzaro, Italy.

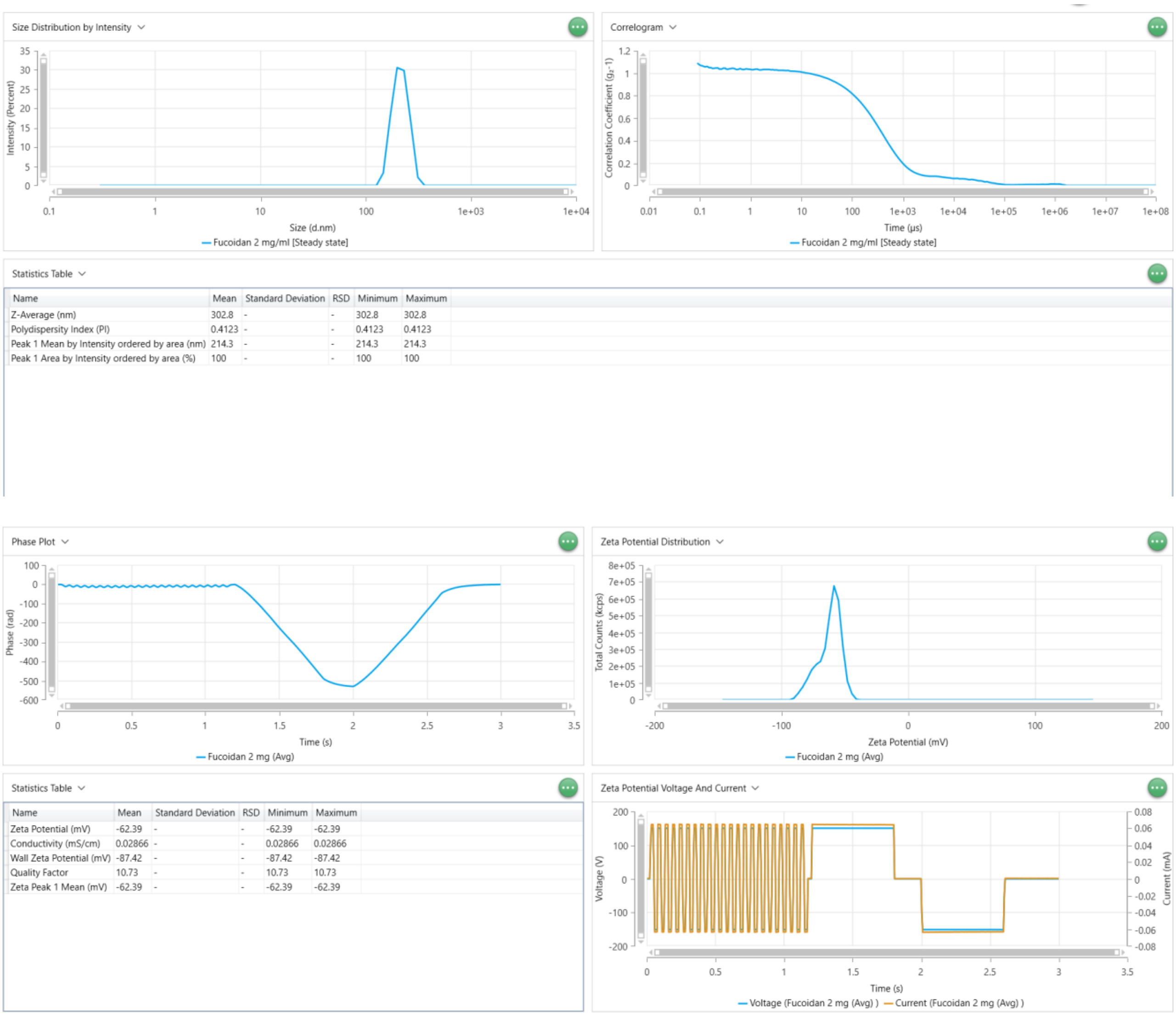

Figure S1. Dynamic Light Scattering datasheet report of nanoparticles prepared with 2 mg/ml of fucoidan.

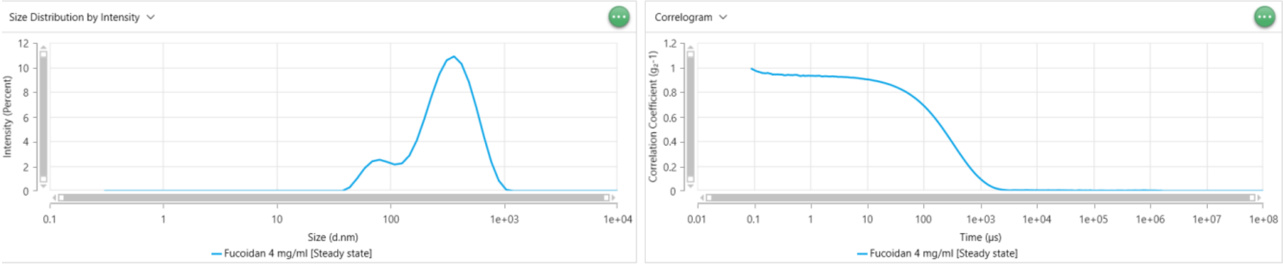

Statistics Table

Name	Mean	Standard Deviation	RSD	Minimum	Maximum
Z-Average (nm)	231.3	-	-	231.3	231.3
Polydispersity Index (PI)	0.2952	-	-	0.2952	0.2952
Peak 1 Mean by Intensity ordered by area (nm)	362.1	-	-	362.1	362.1
Peak 1 Area by Intensity ordered by area (%)	87.64	-	-	87.64	87.64
Peak 2 Mean by Intensity ordered by area (nm)	78.65	-	-	78.65	78.65
Peak 2 Area by Intensity ordered by area (%)	12.36	-	-	12.36	12.36

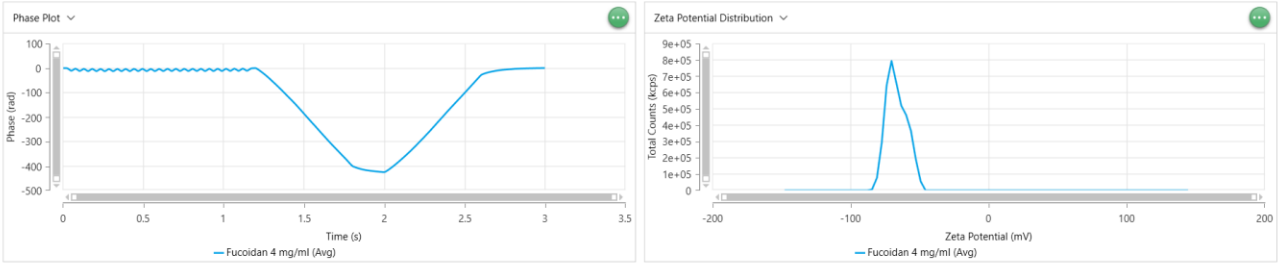

Statistics Table

Name	Mean	Standard Deviation	RSD	Minimum	Maximum
Zeta Potential (mV)	-66.77	-	-	-66.77	-66.77
Conductivity (mS/cm)	0.03237	-	-	0.03237	0.03237
Wall Zeta Potential (mV)	-52.92	-	-	-52.92	-52.92
Quality Factor	10.25	-	-	10.25	10.25
Zeta Peak 1 Mean (mV)	-66.77	-	-	-66.77	-66.77

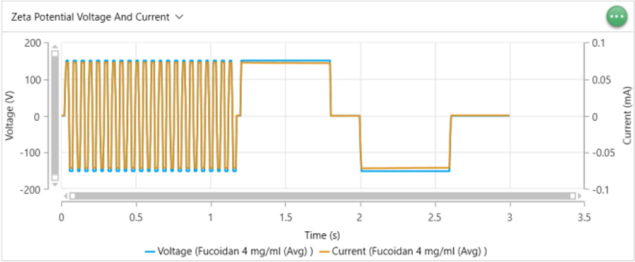

Figure S2. Dynamic Light Scattering datasheet report of nanoparticles prepared with 4 mg/ml of fucoidan.

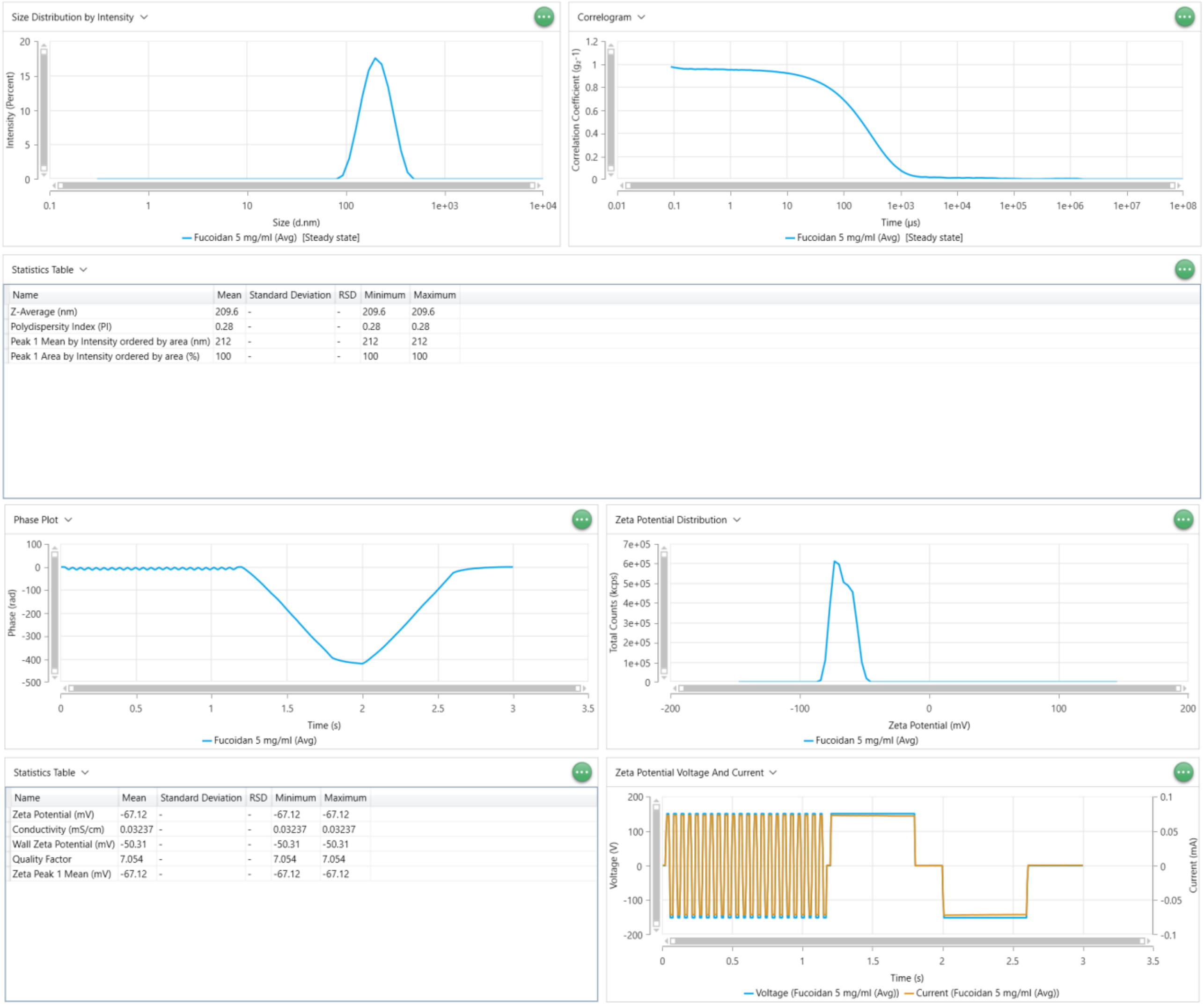

Name	Mean	Standard Deviation	RSD	Minimum	Maximum
Z-Average (nm)	209.6	-	-	209.6	209.6
Polydispersity Index (PI)	0.28	-	-	0.28	0.28
Peak 1 Mean by Intensity ordered by area (nm)	212	-	-	212	212
Peak 1 Area by Intensity ordered by area (%)	100	-	-	100	100

Name	Mean	Standard Deviation	RSD	Minimum	Maximum
Zeta Potential (mV)	-67.12	-	-	-67.12	-67.12
Conductivity (mS/cm)	0.03237	-	-	0.03237	0.03237
Wall Zeta Potential (mV)	-50.31	-	-	-50.31	-50.31
Quality Factor	7.054	-	-	7.054	7.054
Zeta Peak 1 Mean (mV)	-67.12	-	-	-67.12	-67.12

Figure S3. Dynamic Light Scattering datasheet report of nanoparticles prepared with 5 mg/ml of fucoidan.

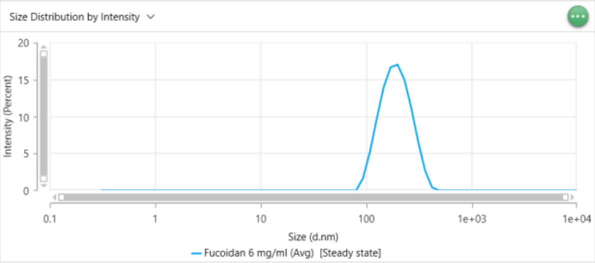

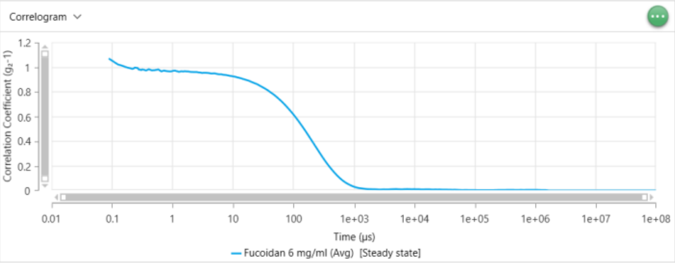

Statistics Table

Name	Mean	Standard Deviation	RSD	Minimum	Maximum
Z-Average (nm)	184.3	-	-	184.3	184.3
Polydispersity Index (PI)	0.2251	-	-	0.2251	0.2251
Peak 1 Mean by Intensity ordered by area (nm)	197.6	-	-	197.6	197.6
Peak 1 Area by Intensity ordered by area (%)	100	-	-	100	100

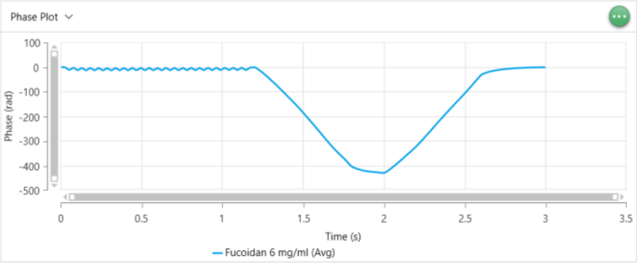

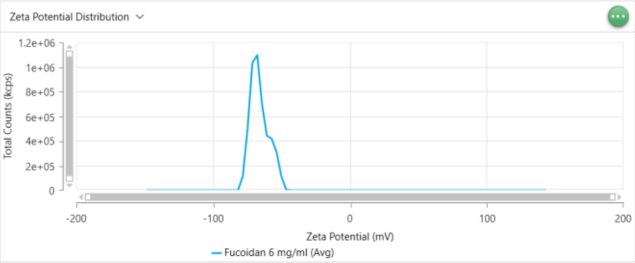

Statistics Table

Name	Mean	Standard Deviation	RSD	Minimum	Maximum
Zeta Potential (mV)	-66.14	-	-	-66.14	-66.14
Conductivity (mS/cm)	0.03237	-	-	0.03237	0.03237
Wall Zeta Potential (mV)	-55.93	-	-	-55.93	-55.93
Quality Factor	7.133	-	-	7.133	7.133
Zeta Peak 1 Mean (mV)	-66.14	-	-	-66.14	-66.14

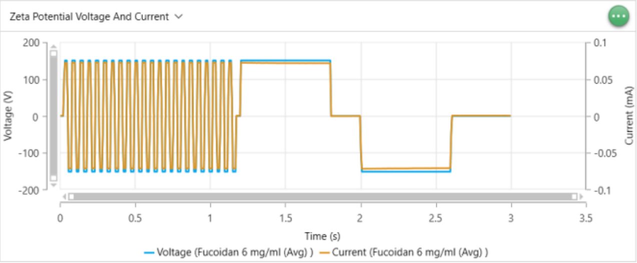

Figure S4. Dynamic Light Scattering datasheet report of nanoparticles prepared with 6 mg/ml of fucoidan.

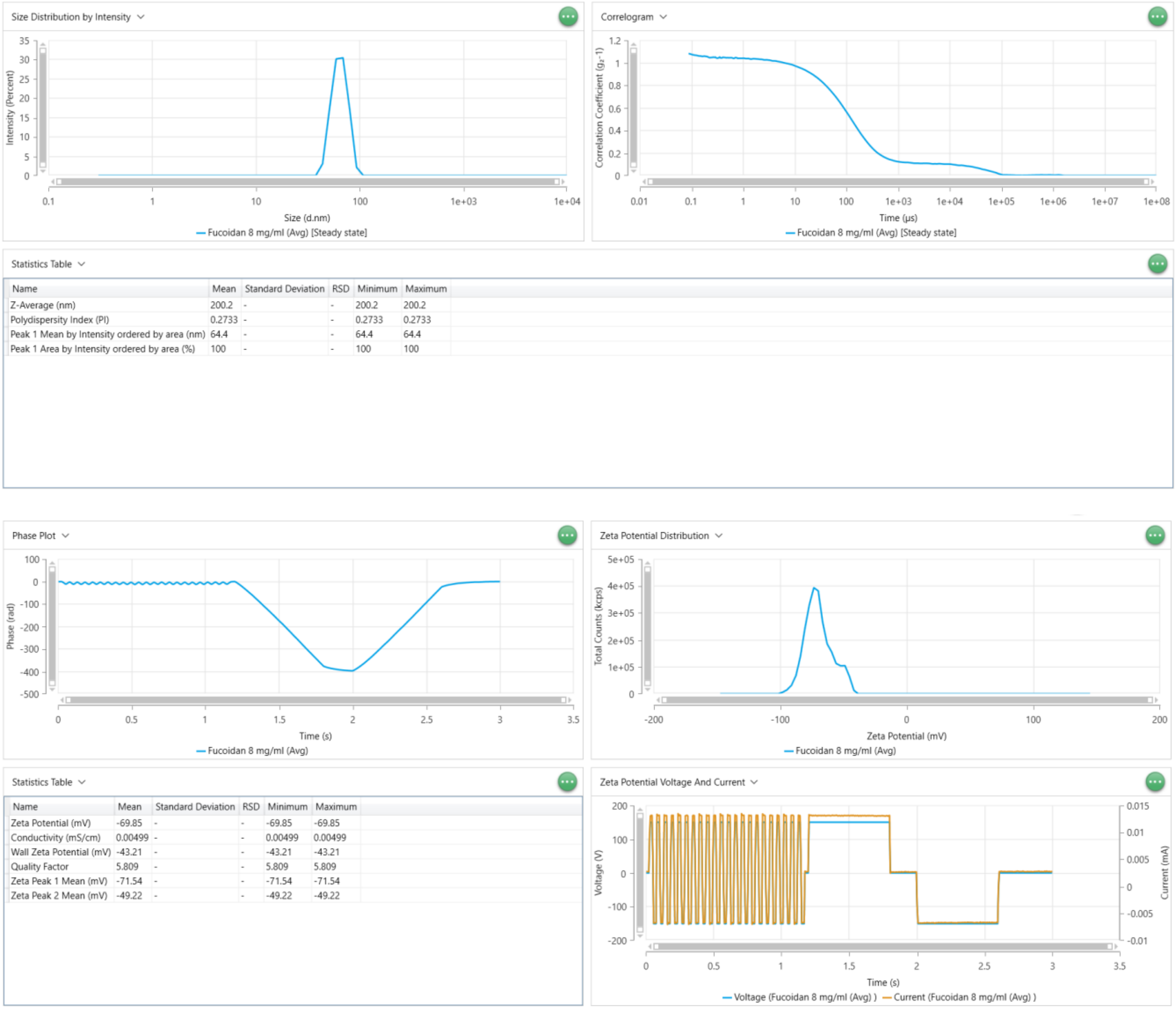

Name	Mean	Standard Deviation	RSD	Minimum	Maximum
Z-Average (nm)	200.2	-	-	200.2	200.2
Polydispersity Index (PI)	0.2733	-	-	0.2733	0.2733
Peak 1 Mean by Intensity ordered by area (nm)	64.4	-	-	64.4	64.4
Peak 1 Area by Intensity ordered by area (%)	100	-	-	100	100

Name	Mean	Standard Deviation	RSD	Minimum	Maximum
Zeta Potential (mV)	-69.85	-	-	-69.85	-69.85
Conductivity (mS/cm)	0.00499	-	-	0.00499	0.00499
Wall Zeta Potential (mV)	-43.21	-	-	-43.21	-43.21
Quality Factor	5.809	-	-	5.809	5.809
Zeta Peak 1 Mean (mV)	-71.54	-	-	-71.54	-71.54
Zeta Peak 2 Mean (mV)	-49.22	-	-	-49.22	-49.22

Figure S5. Dynamic Light Scattering datasheet report of nanoparticles prepared with 8 mg/ml of fucoidan.

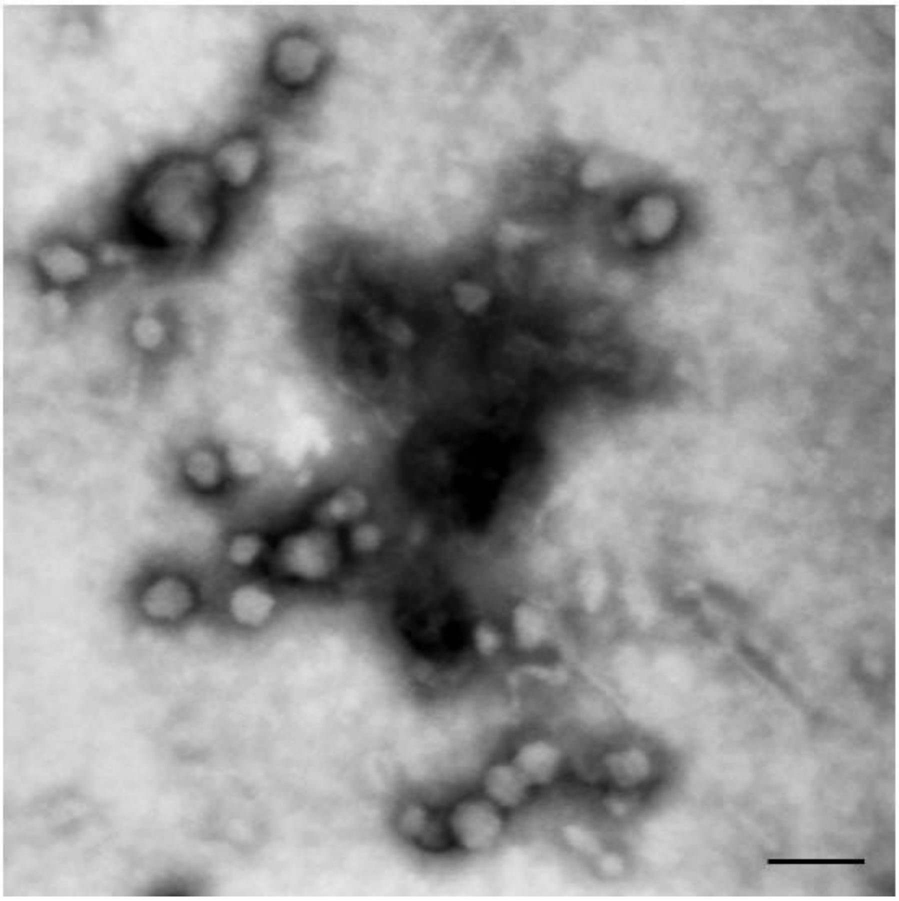

Figure S6. TEM micrograph of fucoidan nanoparticles prepared with 6 mg/ml of biopolymer. Bar = 200 nm.

Cathepsin D

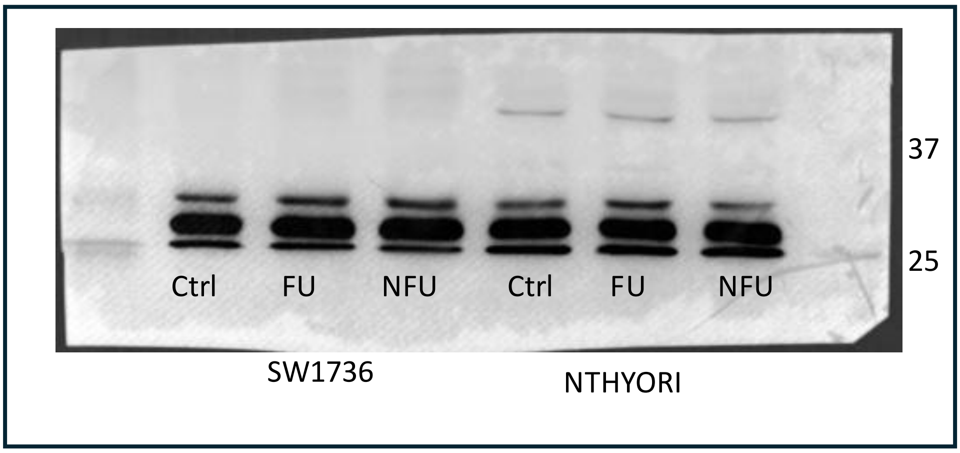

GAPDH

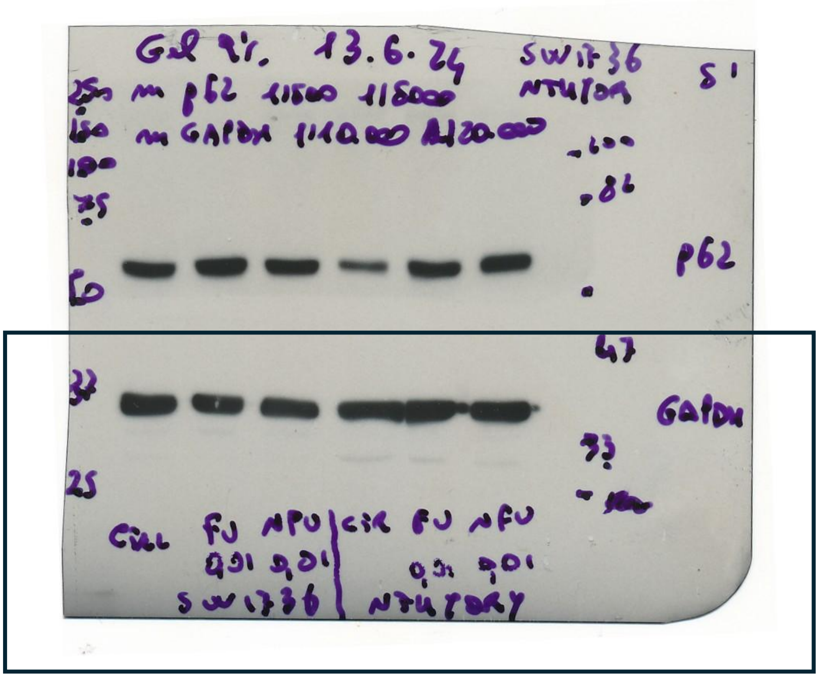

Figure S7. Original uncropped western blotting of images reported in: A, Figure 5
